# Data analytics and clinical feature ranking of medical records of patients with sepsis

**DOI:** 10.1186/s13040-021-00235-0

**Published:** 2021-02-03

**Authors:** Davide Chicco, Luca Oneto

**Affiliations:** 1 Krembil Research Institute, Toronto, Ontario, Canada; 2 Università di Genova, Genoa, Italy; 3 ZenaByte srl, Genoa, Italy

**Keywords:** Sepsis, Septic shock, Septic severity, Survival, Sequential organ failure assessment, SOFA, Machine learning, Binary classification, Regression analysis, Feature ranking, Data science, Data analytics

## Abstract

**Background:**

Sepsis is a life-threatening clinical condition that happens when the patient’s body has an excessive reaction to an infection, and should be treated in one hour. Due to the urgency of sepsis, doctors and physicians often do not have enough time to perform laboratory tests and analyses to help them forecast the consequences of the sepsis episode. In this context, machine learning can provide a fast computational prediction of sepsis severity, patient survival, and sequential organ failure by just analyzing the electronic health records of the patients. Also, machine learning can be employed to understand which features in the medical records are more predictive of sepsis severity, of patient survival, and of sequential organ failure in a fast and non-invasive way.

**Dataset and methods:**

In this study, we analyzed a dataset of electronic health records of 364 patients collected between 2014 and 2016. The medical record of each patient has 29 clinical features, and includes a binary value for survival, a binary value for septic shock, and a numerical value for the sequential organ failure assessment (SOFA) score. We disjointly utilized each of these three factors as an independent target, and employed several machine learning methods to predict it (binary classifiers for survival and septic shock, and regression analysis for the SOFA score). Afterwards, we used a data mining approach to identify the most important dataset features in relation to each of the three targets separately, and compared these results with the results achieved through a standard biostatistics approach.

**Results and conclusions:**

Our results showed that machine learning can be employed efficiently to predict septic shock, SOFA score, and survival of patients diagnoses with sepsis, from their electronic health records data. And regarding clinical feature ranking, our results showed that Random Forests feature selection identified several unexpected symptoms and clinical components as relevant for septic shock, SOFA score, and survival. These discoveries can help doctors and physicians in understanding and predicting septic shock. We made the analyzed dataset and our developed software code publicly available online.

**Supplementary Information:**

The online version contains supplementary material available at (10.1186/s13040-021-00235-0).

## Background

Sepsis is a dangerous clinical condition that happens when the body over-reacts to an infection, and its mortality is strictly related to sepsis severity. The more severe is the sepsis, the more risks there are for the patient.

Predicting the severity of a sepsis episode and if a patient will survive it are urgent tasks, because of the riskiness of this condition. A severe sepsis episode is called *septic shock*. Septic shocks require the prompt use of vasopressors, and must be treated immediately to improve the survival chances of the patient [1].

In addition to sepsis severity and survival prediction, another important task for doctors and physicians is to anticipate the possible sequential organ failure assessment that the patient will experience as a consequence of the sepsis episode. To diagnose the level of organ failure happening in the body, the biomedical community takes advantage of the sequential organ failure assessment (SOFA) score [1], which is based upon six different rates (respiratory, cardiovascular, hepatic, coagulation, renal and neurological systems) [1].

In this context, machine learning and artificial intelligence applied to electronic health records (EHRs) of patients diagnosed with sepsis can provide cheap, fast, non-invasive and effective methods that are able to predict the aforementioned targets (septic shock, survival, and SOFA score), and to detect the most predictive symptoms and risk factors from the features available in the electronic health records. Scientists, in fact, already took advantage of machine learning for survival or diagnosis prediction and for clinical feature ranking several times in the past [2], for example to analyze datasets of patients having heart failure [3, 4], mesothelioma [5], neuroblastoma [6–8], and breast cancer [9].

Several researchers employed computational intelligence algorithms to medical records of patients diagnosed with sepsis, too, especially for clinical decision-making purposes.

Gultepe and colleagues [10] applied machine learning to the EHRs of 741 adults diagnosed with sepsis at the University of California Davis Health System (California, USA) to predict lactate levels and mortality risk of the patients. Tsoukalas et al. [11] employed several pattern recognition algorithms to analyze medical record data of 1,492 patients diagnosed with sepsis at the same health centre. Their data-derived antibiotic administration policies improved the conditions of patients. Taylor and colleagues [12] analyzed medical records of a cohort of approximately 260 thousand individuals from three hospitals in the USA. They used machine learning to predict in-hospital mortality of patients diagnosed with sepsis, and to show the superior results of machine learning over traditional univariate biostatistics techniques. Horng et al. [13] applied computational intelligence techniques to medical records of 230,936 patient visits containing heterogeneous data: free text, vital signs, and demographic information. The dataset was collected at the Beth Israel Deaconess Medical Center (BIDMC) of Boston (Massachusetts, USA). Shimabukuro and colleagues [14] employed machine learning techniques to clinical records of 142 patients with severe sepsis from University of California San Francisco Medical Center (California, USA) to predict the in-hospital length of stay and mortality rate. Burdick et al. [15] used several computational intelligence methods on medical records of 2,296 patients related to sepsis, that were provided by Cabell Huntington Hospital (Huntington, West Virginia, USA). Their goal was to predict patients’ mortality and in-hospital length of stay. Calvert and colleagues [16] merged together several datasets of clinical records of sepsis-related patients to create a large cohort of approximately 500 thousand individuals. Then they used machine learning to forecast how the high-risk patients are likely to have a sepsis episode. Barton et al. [17], lastly, re-analyzed two datasets previously exploited [13, 14] to predict sepsis up to 48 hours in advance.

Scientists employed machine learning for the prediction of sepsis in infants in the neonatal intensive care unit (NICU), as well. In 2014, Mani and colleagues [18] applied nine machine learning methods to 299 infants admitted to the neonatal intensive care unit in the Monroe Carell Junior Children’s Hospital at Vanderbilt (Nashville, Tennessee, USA). Barton et al. [19] took advantage of data mining classifiers to analyze the EHRs of 11,127 neonatal patients collected at the University of California San Francisco Medical Center (California, USA). More recently, Masino and his team [20] applied computational intelligence classifiers to the data of infants admitted at the neonatal intensive care unit of the Children’s Hospital of Philadelphia (Pennsylvania, USA).

To recap, four studies applied machine learning to minimal electronic health records to diagnose sepsis or predict survival of patients [10, 11, 21, 22], while six other studies applied them to complete electronic health records for the same goals [12–14, 16, 17, 19]. The study of Burdick and colleagues [15] even reported an observed decreased in the mortality at the hospital where the computational intelligence methods were applied to recognize sepsis. Only two articles, additionally, include a feature ranking phase to the binary classification: Mani et al. [18] identified as most predictive variables hematocrit or packed cell volume, chorioamnionitis and respiratory rate, while Masino and coauthors [20] highlighted central venous line, mean arterial pressure, respiratory rate difference, systolic blood pressure.

Our present study fits in the latter category: we use several machine learning methods not only to predict survival, SOFA score, and septic shock, but also to detect the most relevant and predictive variables from the electronic health records. Moreover, we also perform a feature ranking through traditional biostatistics rates, and make a comparison between the results obtained with these two different approaches. And, differently from all the studies mentioned earlier, we do not focus only on predicting survival and diagnosing sepsis, but we also make computational predictions on the SOFA score, that means predicting how much and how many organs will fail because of the septic episode.

Regarding scientific challenges and competitions, in 2019 PhysioNet [23, 24], an online platform for physiologic data sharing, launched an online scientific challenge for the prediction of early sepsis in medical records [25].

On the business side, the San Francisco bay area startup company Dascena Inc. recently released *InSight*, a machine learning tool able to computational predict sepsis in EHR data [26]. Desautels et al. [21] applied *InSight* to predict sepsis in the medical records of the Multiparameter Intelligent Monitoring in Intensive Care (MIMIC)-III dataset [27].

In the present study, we analyzed a dataset of electronic health records of patients having cardiovascular heart diseases [28]: each patient profile has 29 clinical features, including a binary value for survival, a binary value for septic shock, and a numerical value for the sequential organ failure assessment (SOFA) score. We separately used each of these three features as an independent target, and employed several machine learning classifiers to predict it with high accuracy and precision. Afterwards, we employed machine learning to detect the most important features of the dataset for the three target separately, and compared its results with the results obtained through traditional biostatistics univariate techniques.

## Dataset

The original dataset contains electronic health records (EHRs) of 29 features for 364 patients, and was first analyzed by Yunus and colleagues to investigate the role of procalcitonin in sepsis [29]. These 364 patients with sepsis diagnosis entered the general medical ward and intensive care unit between September 2014 and December 2016 at the Methodist Medical Center and Proctor Hospital (today called UnityPoint Health – Methodist ∣ Proctor) in Peoria, Illinois, USA [29]. The group of patients include 189 men and 175 women, aged 20–86 years old [29, 30].

Each patient stayed at the hospital for a period between 1 and 48 days, and her/his dataset profile represent the co rresponding clinical record at the moment of discharge or death. Since the maximum observation window was 48 days, we consider our binary predictions in reference to the same time frame.

The dataset collectors defined septic shock “as a condition that requires the use of vasopressors in order to maintain a mean arterial pressure (MAP) of 65 mm Hg or above, and a persistent lactate greater than 2 mmol/L in spite of adequate fluid resuscitation” [29, 30].

We report the quantitative characteristics of the dataset (amount of individuals and percentage of individuals for each binary feature condition; median and mean for each numeric or category feature) in Table 1, and the interpretation details (meaning, measurement unit, and value range in the dataset) in Table 2. More information about the analyzed dataset can be found in the original dataset curators publication [29, 30].

**Table 1:** Statistical quantitative description of the features. Binary and category features on the left, and numeric features on the right

binary feature	#	%	numeric feature	median	mean
atrial fibrillation (1: yes)	49	13.46	age	63	61.32
atrial fibrillation (2: no)	315	86.54	anatomical site of infection	2	2.84
cancer (1: yes)	71	19.51	bilirubin	0.6	0.87
cancer (2: no)	293	80.49	creatinine	1.37	1.96
chronic kidney disease (CKD) with dialysis (1: yes)	16	4.40	extent of infection	2	1.80
chronic kidney disease (CKD) with dialysis (2: no)	348	95.60	Glasgow coma scale	15	13.45
chronic kidney disease (CKD) without dialysis (1: yes)	51	14.01	initial procalcitonin (PCT)	1.65	13.88
chronic kidney disease (CKD) without dialysis (2: no)	313	85.99	mean arterial pressure	79	79.68
chronic obstructive pulmonary disease (COPD) (1: yes)	109	29.95	microorganism	2	2.32
chronic obstructive pulmonary disease (COPD) (2: no)	255	70.05	platelets	216	231.16
congestive heart failure (CHF) (1: yes)	72	19.78	respiration (PaO2)	79	93.86
congestive heart failure (CHF) (2: no)	292	80.22	respiration (FiO2)	32	45.79
coronary artery disease (CAD) (1: yes)	81	22.25	urine output 24 hours	1	1.14
coronary artery disease (CAD) (2: no)	283	77.75	[target] SOFA score	4	5.06
diabetes mellitus (DM) (1: yes)	135	37.09			
diabetes mellitus (DM) (2: no)	229	62.91			
hypertension (HTN) (1: yes)	152	41.76			
hypertension (HTN) (2: no)	212	58.24			
mechanical vent (1: yes)	97	26.65			
mechanical vent (2: no)	267	73.35			
pulmonary embolism (PE) (1: yes)	6	1.65			
pulmonary embolism (PE) (2: no)	358	98.35			
sex (1: male)	175	48.08			
sex (2: female)	189	51.92			
[target] survival (0: no)	48	13.19			
[target] survival (1: yes)	316	86.81			
[target] vasopressors (0: no)	67	18.41			
[target] vasopressors (1: yes)	297	81.59

**Table 2:** Dataset feature description. Meanings, measurement units, and intervals of each feature of the dataset

feature: explanation	measurement unit	range
age: age of the patient	years	[20,..., 86]
anatomical site of infection: body location	category	[1, 2, 3,..., 16]
atrial fibrillation: presence	boolean	[1, 2]
bilirubin: level in blood	mg/dL	[0.10,..., 22.50]
cancer: presence	boolean	[1, 2]
chronic kidney disease (CKD) with dialysis: presence	boolean	[1, 2]
chronic kidney disease (CKD) without dialysis: presence	boolean	[1, 2]
chronic obstructive pulmonary disease (COPD): presence	boolean	[1, 2]
congestive heart failure (CHF): presence	boolean	[1, 2]
coronary artery disease (CAD): presence	boolean	[1, 2]
creatinine: level in blood	mg/dL	[0.15,..., 15.10]
diabetes mellitus: presence	boolean	[1, 2]
extent of infection: type of infection	category	[1, 2, 3]
Glasgow coma scale: neurological scale to measure coma	category	[2, 3,..., 15]
hypertension (HTN): presence	boolean	[1, 2]
initial procalcitonin (PCT): level in blood	ng/mL	[0.05,..., 252.50]
mean arterial pressure: blood pressure during single cardiac cycle	mm Hg	[9, 44,..., 138]
mechanical vent: if the patient needs mechanical ventilation	boolean	[1, 2]
microorganism: kind of bacterial infection	category	[1, 2,..., 6]
platelets: level in blood	kilo/microL	[3.0,..., 726.0]
pulmonary embolism: presence	boolean	[1, 2]
respiration (PaO2): partial pressure of oxygen	mm Hg	[2, 21,..., 595]
respiration (FiO2): fraction of inspired oxygen	mm Hg	[21, 25,..., 262]
sex: woman or man	boolean	[1, 2]
urine output 24 hours: patient’s urine in the day	category (mL/24 hours)	[1, 2, 3]
[target] SOFA score: sequential organ failure assessment score	category	[0, 1, 2,..., 23]
[target] survival: survival or death	boolean	[0, 1]
[target] vasopressors septic shock: presence	boolean	[0, 1]

We derived the survival feature from the outcome feature of the original dataset (Supplementary information) [31]. The extent of the infection feature can have 3 values that represent bacteremia, focal infection, or both. The urine output 24 hours feature can have 3 values that represent >500 mL, [200,500] mL, or <200 mL.

Regarding the dataset imbalance, considering septic shock as the target, there are 297 individuals without septic shock (having value 0 for the vasopressors feature), corresponding to 81.59% of the total size, and 67 individuals with septic shock (having value 1 for the vasopressors feature), corresponding to 18.41% of the total size.

When we consider the survival as target, instead we observe 48 deceased patients (class 0, corresponding to 13.19% of all the individuals), and observe 316 survived patients (class 1, corresponding to 86.81% of all the individuals).

The dataset with septic shock as target results therefore negatively imbalanced, and the dataset with survival as target results positively imbalanced.

## Methods

We implemented our computational pipeline in the open-license, free R programming language, using common machine learning packages (randomForest, caret, e1071, keras, ROSE, DMwR, mltools, DescTools). We also released all our code scripts publicly online (“Availability of data and materials”).

As described in subsection S7.2, we can recap the computation pipeline of the analsys with the following steps:

1. construction of the dataset (“Dataset” section);
2. definition of the three tasks:
  a the binary classification problem of predicting septic shock (vasopressors);
  b the regression problem of predicting SOFA score;
  c the binary classification problem of predicting survival;
  based on a subset of the available variables selected as input variables (Table 2);
3. for each of these three tasks (septic shock, survival, and SOFA score) and for each of the algorithms (*DT*, *RF*, *SVM (linear)*, *SVM (kernel)*, and *NN*, (*DT*, *RF*, *SVM (linear)*, *SVM (kernel)*, *NB*, *k-NN*, *LR*, and *DL*, noting that NB and LR can be used just for classification problems) we built a model using the MS strategy (“Methods” section) where we set the number of fold *k*=10. During the MS we searched the hyper-parameters using the following ranges
  a *DT*: $\mathcal {H} = \{ d \} \in \{ 2, \, 4, \, 6, \, 8, \, 10, \, 12, \, 14 \}$;
  b *RF*: we set *n*_*t*_=1000 since increasing it does not increases the accuracy;
  c *SVM (linear)*: $\mathcal {H} = \{ C \} \in \mathcal {R}$;
  d NB: we use kernel density estimate no Laplace correction and no adjustment (R library caret*nb* algorithm);
  e *k*-NN: $\mathcal {H} = \{ k \} \in \{ 1,3,5,11 \}$;
  f LR: $\mathcal {H} = \{ \lambda \} \in \mathcal {R}$;
  g DL: $\mathcal {H} = \{ l_{1}, l_{2}, l_{3}, wd \} \in \{ 2,4,8,16,32 \} \times \{ 2,4,8,16,32 \} \times \{ 2,4,8,16,32 \} \times \{.001,.01,.1,1 \} $ ;
  h *SVM (kernel)*: $\mathcal {H} = \{ C, \gamma \} \in \mathcal {R} \times \mathcal {R}$;
  i *NN*: $\mathcal {H} = \{ n_{h}, \, p_{d}, \, p_{b}, \, r_{l}, \, \rho, \, r_{d} \} \in \{ 5, \, 10, \, 20, \, 40, \, 80, \, 160 \} \times \{ 0, \, 0.001, \, 0.01, \, 0.1 \} \times \{ 0.1, \, 1 \} \times \{ 0.001,0.01,0.1,1 \} \times \{ 0.9,0.09 \} \times \{ 0.001,0.01,0.1,1 \}$ and as activation function we used the rectified linear unit (ReLU) [32];
  where $\mathcal {R} = \{ 0.0001, \, 0.0005, \, 0.001, \, 0.005, \, 0.01, \, 0.05, \, 0.1, \, 0.5, \, 1, \, 5, \, 10, \, 50 \}$;
4. for each of the constructed models we reported the results using the EE strategy and previously introduced the metrics (“Methods” section) together with the standard deviation where we set *n*_*r*_=100;
5. for each of the tasks we reported the ranking of the features selected by the two feature ranking procedures (*MDI* and *MDA*, “Methods” section) together with the mode of the ranking position where we set *p*_FR_=0.7 and *n*_FR_=100, and aggregated through Borda’s method [33].

We report and discuss the results in the next sections.

## Results

In this section we show the results of applying the classification and regression methods (“Methods” section) on the described dataset (“Dataset” section).

### Target predictions

In this section, we describe the results obtained for the binary prediction of septic shock, for the SOFA score regression estimation, and for the binary prediction of survival in the ICU. For the two binary classifications (septic shock prediction and survival prediction), we used *τ*=0.5 as cut-off threshold for the confusion matrices. We chose this value because it corresponds to the value 0 for the Matthews correlation coefficient (MCC) [34], which means the predicted value is not better than random prediction.

We focused on and ordered our results by the scores of the MCC, because this rate provides a high score only if the classifier was able to correctly predict the majority of positive data instances and the majority of the negative data instances, despite of the dataset imbalance [35, 36].

In the interest of providing fuller information, we also reported the values of ROC AUCs [37] and PR AUCs [38], which are computed considering all the possible confusion matrix thresholds.

#### Septic shock prediction

We report the performance of the learned models for the septic shock (vasopressors) prediction with the different methods evaluated with the different metrics in Table 3, ranked by the MCC.

**Table 3:** Septic shock (vasopressors) prediction

method	MCC	F_1_ score	accuracy	TP rate	TN rate
RF	**0*.*32±0*.*14**	0.88±0.03	0.80±0.04	0.88±0.04	0.43±0.15
MLP	**0*.*31±0*.*13**	0.87±0.03	0.79±0.04	0.87±0.04	0.47±0.15
LR	**0*.*31±0*.*13**	0.84±0.04	0.76±0.05	0.79±0.06	0.62±0.15
DL	**0*.*30±0*.*11**	0.83±0.05	0.73±0.04	0.78±0.05	0.61±0.16
NB	**0*.*27±0*.*08**	0.79±0.09	0.70±0.09	0.72±0.15	0.59±0.18
SVM (linear)	**0*.*26±0*.*13**	0.82±0.06	0.75±0.06	0.82±0.09	0.49±0.18
*k*-NN	**0*.*23±0*.*14**	0.81±0.06	0.71±0.07	0.76±0.10	0.50±0.20
SVM (kernel)	**0*.*22±0*.*13**	0.79±0.06	0.70±0.06	0.75±0.09	0.50±0.18
DT	**0*.*18±0*.*13**	0.78±0.06	0.67±0.07	0.72±0.10	0.50±0.19
method	PR AUC	ROC AUC	PPV	NPV	
RF	0.18±0.07	0.28±0.29	0.47±0.16	0.87±0.05	
MLP	0.16±0.05	0.28±0.28	0.46±0.16	0.87±0.05	
LR	0.11±0.04	0.26±0.31	0.41±0.12	0.90±0.04	
DL	0.11±0.04	0.26±0.31	0.39±0.12	0.88±0.04	
NB	0.11±0.06	0.26±0.29	0.34±0.09	0.89±0.04	
SVM (linear)	0.15±0.05	0.26±0.24	0.37±0.13	0.86±0.06	
*k*-NN	0.13±0.06	0.25±0.30	0.33±0.12	0.87±0.06	
SVM (kernel)	0.13±0.05	0.24±0.20	0.32±0.11	0.86±0.06	
DT	0.12±0.05	0.23±0.29	0.29±0.10	0.86±0.06	

Performance of the learned models with the different methods evaluated with the different metrics, expressed in the format “average value ± standard deviation”, obtained on 100 executions. DT: decision tree. MLP: multi-layer perceptron neural network. RF: random forest. *k*-NN: k-nearest neighbors. DL: deep neural network with 3 hidden layers and weight decay. LR: logistic regression. NB: Naïve Bayes. SVM (kernel): support vector machine with kernel. SVM (linear): linear support vector machine. MCC: Matthews correlation coefficient. TP rate: true positive rate (sensitivity, recall). TN rate: true negative rate (specificity). PR: precision-recall curve. ROC: receiver operating characteristic. AUC: area under the curve. MCC: worst value –1.00 and best value +1.00. PPV: positive predictive value (precision). NPV: negative predictive value. F_1_ score, accuracy, TP rate, TN rate, PR AUC, ROC AUC, PPV, NPV: worst value 0.00 and best value 1.00. Imbalance of this dataset: yes septic shock class: 1’s positives, #elements = 67 (18.41%), and no septic shock class: 0’s negatives, #elements = 297 (81.59%)

Our methods were able to obtain high prediction results and showed the ability of machine learning to predict septic shock (positive data instances), but showed low ability to identify patients without septic shock (negative data instances). In particular, Random Forests and the Multi-Layer Perceptron Neural Network outperformed the other methods (Table 3), by achieving average MCC equal to +0.32 and +0.31, respectively. All the classifiers obtained high scores for the true positive rate, accuracy, and F_1_ score, but achieved low scores on the true negative rates (Table 3). Decision Tree, kernel SVM, Logistic Regression, Deep Learning, and Naive Bayes were the only methods which predicted correctly most of the negative instances, by achieving average specificity equal to 0.50.

Regarding ROC AUC, it is interesting to notice that standard deviations for all the methods is high (standard deviation from 0.20 to 0.31. Table 3).

To check the predictive efficiency of the algorithms in making positive calls, we reported the positive predictive value (PPV, or *precision*). From a clinical perspective, the PPV represents the likelihood that patients with a positive screening test truly have the septic shock [39]. The PPV results show that Random Forests achieved the top performance among the methods tried, but was unable to correctly make the majority of the positive calls (PPV=0.47 in Table 3). This result means that, for each patient predicted to have septic shock, we cannot be sure that she/he will actually have a septic shock: there is an average top probability of 47% that she/he might have it, which leaves large room for uncertainty.

From a clinical perspective, the negative predictive value (NPV) represents the probability that a patient who got a negative screening test will truly not suffer from a septic shock [39]. Regarding this ratio of correct negative predictions, all the methods achieved good results, with Logistic Regression outperforming the other ones (NPV=0.90 in Table 3). This result means that, for each patient predicted not to have septic shock, we can be at 90% confident that he/she will not have septic shock, which leaves small room for uncertainty.

#### SOFA score prediction

**Fig. 1:**
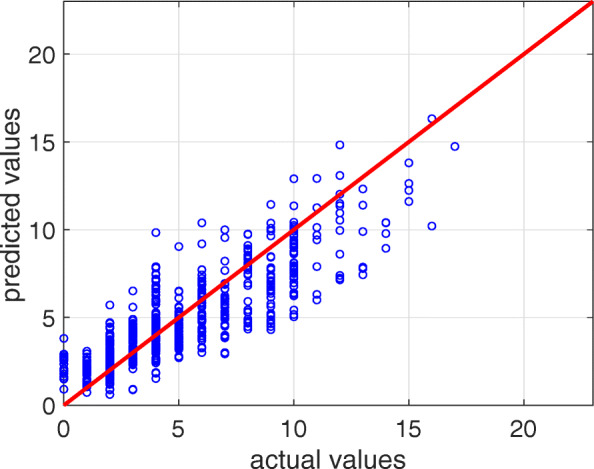
SOFA score regression. Scatterplot of the actual and predicted value of the *n*_*e*_ test sets for the SVM (linear) method

We report the performance of the learned models for the SOFA score prediction with the different methods evaluated with the different metrics in Table 4, ordered by the coef- ficient of determination R^2^, and the SOFA score scatterplot of the actual and predicted value of the *n*_*e*_ test sets in Fig. 1. We used R^2^ for the method sorting because this rate incorporates the SOFA score distribution.

**Table 4:** SOFA score prediction

method	R^2^	RMSE	MAE	MSE	SMAPE
DL	**0*.*73±0*.*05**	1.85±0.26	1.31±0.12	3.33±0.99	0.34±0.04
SVM (linear)	**0*.*78±0*.*06**	1.81±0.18	1.34±0.13	3.27±0.60	0.34±0.05
MLP	**0*.*76±0*.*06**	1.82±0.13	1.36±0.11	3.29±0.71	0.34±0.04
SVM (kernel)	**0*.*74±0*.*06**	1.82±0.25	1.35±0.13	3.37±1.06	0.33±0.03
RF	**0*.*72±0*.*04**	1.83±0.26	1.32±0.15	3.41±1.01	0.35±0.03
DT	**0*.*48±0*.*11**	2.46±0.28	1.80±0.18	6.11±1.38	0.49±0.07
*k*-NN	**0*.*41±0*.*10**	2.65±0.35	1.95±0.22	7.12±1.85	0.53±0.06

Performance of the learned models with the different methods evaluated with the different metrics, expressed in the format “average value ± standard deviation”, obtained on 100 executions. DT: decision tree. *k*-NN: k-nearest neighbors. DL: deep neural network with 3 hidden layers and weight decay. MLP: multi-layer perceptron neural network. RF: random forest. SVM (kernel): support vector machine with kernel. SVM (linear): linear support vector machine. RMSE: root mean square error. MAE: mean absolute error. MSE: mean square error. SMAPE: symmetric mean absolute percentage error. R^2^: coefficient of determination. RMSE, MAE, MSE, SMAPE: best value 0.00 and worst value +*∞*. R^2^: best value 1.00 and worst value −*∞*

Our results show that machine learning can predict SOFA score with low error rates (Table 4). Differently from the septic shock prediction, here the Deep Learning model resulted as the top classifier by outperforming the other methods in the R^2^ and MAE. The linear SVM, the Multi-Layer Perceptron, the kernel SVM, and Random Forests obtained similar results, and resulted in being close to the top method for this task. It is interesting to notice that linear SVM resulted in being the top method when its predictions were measured through RMSE and MSE, but not in the other cases.

#### Survival prediction

We report the performance of the learned models for the survival prediction with the different methods evaluated with the different metrics in Table 5, ranked by the MCC.

**Table 5:** Survival prediction

method	MCC	F_1_ score	accuracy	TP rate	TN rate
MLP	**0*.*31±0*.*12**	0.43±0.11	0.67±0.08	0.75±0.12	0.70±0.07
DL	**0*.*16±0*.*10**	0.30±0.07	0.72±0.05	0.50±0.12	0.82±0.07
NB	**0*.*15±0*.*11**	0.28±0.08	0.82±0.05	0.21±0.12	0.92±0.06
RF	**0*.*15±0*.*10**	0.28±0.08	0.64±0.05	0.58±0.16	0.65±0.06
LR	**0*.*13±0*.*12**	0.26±0.08	0.69±0.05	0.45±0.20	0.73±0.06
SVM (linear)	**0*.*11±0*.*13**	0.26±0.12	0.72±0.09	0.46±0.19	0.71±0.11
DT	**0*.*10±0*.*12**	0.25±0.11	0.69±0.08	0.39±0.17	0.74±0.09
SVM (kernel)	**0*.*09±0*.*11**	0.24±0.09	0.72±0.06	0.38±0.16	0.75±0.08
*k*-NN	**0*.*08±0*.*13**	0.22±0.09	0.69±0.11	0.38±0.24	0.73±0.15
method	PR AUC	ROC AUC	PPV	NPV	
MLP	0.29±0.07	0.86±0.13	0.94±0.07	0.19±0.07	
DL	0.20±0.06	0.86±0.18	0.88±0.05	0.19±0.13	
NB	0.07±0.06	0.80±0.28	0.88±0.03	0.31±0.16	
RF	0.32±0.06	0.86±0.25	0.91±0.04	0.19±0.07	
LR	0.24±0.05	0.86±0.24	0.90±0.04	0.20±0.08	
SVM (linear)	0.27±0.11	0.83±0.24	0.90±0.04	0.19±0.08	
DT	0.23±0.08	0.90±0.23	0.89±0.04	0.19±0.09	
SVM (kernel)	0.20±0.07	0.80±0.24	0.89±0.04	0.19±0.07	
*k*-NN	0.23±0.13	0.91±0.21	0.89±0.05	0.18±0.11	

Performance of the learned models with the different methods evaluated with the different metrics, expressed in the format “average value ± standard deviation”, obtained on 100 executions. DT: decision tree. *k*-NN: k-nearest neighbors. DL: deep neural network with 3 hidden layers and weight decay. LR: logistic regression. NB: naive Bayes. MLP: multi-layer perceptron neural network. SVM (kernel): support vector machine with kernel. SVM (linear): linear support vector machine. MCC: Matthews correlation coefficient. TP rate: true positive rate (sensitivity, recall). TN rate: true negative rate (specificity). PR: precision-recall curve. ROC: receiver operating characteristic. AUC: area under the curve. MCC: worst value –1.00 and best value +1.00. PPV: positive predictive value (precision). NPV: negative predictive value. F_1_ score, accuracy, TP rate, TN rate, PR AUC, ROC AUC, PPV, NPV: worst value 0.00 and best value 1.00. Imbalance of this dataset: survived patients’ class: 1’s positives, #elements = 316 (86.81%), and deceased patients’ class: 0’s negatives, #elements = 48 (13.19%)

Our results show that it is possible to use machine learning to predict the survival of sepsis patients, with high accuracy (Table 5). In this case, the MLP neural network outperformed the other classifiers by obtaining higher scores for MCC, F_1_ score, and true positive rate. All the methods obtained high results on the true negative rates, but only the MLP neural network and Random Forests were able to predict most of the positive data instances, obtaining average sensitivity equal to 0.75 and 0.58, respectively.

Regarding correct positive predictions (PPV), all the methods were able to correctly make positive predictions (Table 5), while they obtained low results for the ratio of correct negative predictions (NPV).

Contrarily to what happened previously for the septic shock (“Septic shock prediction” section), here we can be confident that the patients predicted to survive will actually survive (top PPV=0.94 for MLP). However, the low NPV values state that the probability of decease of patient predicted as “non survival” is just 0.31%on average for the best method (Naive Bayes), making our predictions less trustworthy in this case.

### Feature rankings

In this section, we present the feature ranking results for the three targets (septic shock, SOFA score, and survival), obtained through Random Forests and through traditional univariate biostatistics approaches.

For complete information, we reported the feature rankings measured thorugh Random Forests as barcharts in the Supplementary Information (Figure S3, Figure S4, andFigure S2).

#### Septic shock feature ranking

We reported the feature ranking for the septic shock obtained by the two feature selections performed through Random Forests (Methods) in Table 6, and the feature rankings obtained through traditional biostatistics coefficients (Pearson correlation coefficient, Student’s *t*-test, *p*-values) in Table 7.

**Table 6:** Septic shock (vasopressors) feature ranking – Random Forests

position	MDI	MDA	feature
1	4.73	7.48×10^−03^	creatinine
2	3.19	1.13×10^−02^	Glasgow coma scale
3	4.72	9.22×10^−03^	mean arterial pressure
4	4.70	6.19×10^−03^	initial procalcitonin (PCT)
5	3.43	1.89×10^−03^	platelets
6	3.14	4.74×10^−03^	anatomical site of infection
7	2.60	5.87×10^−03^	respiration (FiO2)
8	2.86	1.90×10^−03^	respiration (PaO2)
9	1.42	5.74×10^−03^	mechanical vent
10	2.81	7.43×10^−04^	age***
11	1.37	1.13×10^−03^	microorganism
12	6.71×10^−01^	1.01×10^−03^	urine output 24 hours
13	4.92×10^−01^	1.82×10^−04^	hypertension (HTN)
14	3.32×10^−01^	7.19×10^−04^	chronic kidney disease (CKD) without dialysis
15	3.82×10^−01^	2.72×10^−04^	coronary artery disease (CAD)
16	2.36	1.07×10^−04^	bilirubin
17	3.46×10^−01^	3.16×10^−04^	extent of infection
18	4.02×10^−01^	−3.68×10^−05^	diabetes mellitus (DM)
19	3.14×10^−01^	2.06×10^−05^	congestive heart failure (CHF)
20	2.12×10^−01^	5.70×10^−04^	chronic kidney disease (CKD) with dialysis
21	4.32×10^−01^	−3.62×10^−04^	sex
22	2.21×10^−01^	−3.58×10^−06^	atrial fibrillation
23	3.49×10^−01^	−1.00×10^−04^	chronic obstructive pulmonary disease (COPD)
24	3.08×10^−01^	−1.12×10^−04^	cancer
25	3.69×10^−02^	−1.28×10^−05^	pulmonary embolism (PE)

Feature ranking results obtained through Random Forests. ***: feature in a ranking position that clearly differs from its ranking positions in all the biostatistics analysis feature rankings (Table 7)

**Table 7:** Septic shock (vasopressors) features ranking – biostatistics analysis

position	abs(t) rank	abs(t)	*p*-value rank	*p*-value	abs(PCC) rank	abs(PCC)
1	age	89.81	age	2.49×10^−250^	Glasgow coma scale	0.29
2	mean arterial pressure	86.51	mean arterial pressure	1.55×10^−244^	initial procalcitonin (PCT)	0.28
3	Glasgow coma scale	73.55	Glasgow coma scale	5.62×10^−225^	mechanical vent	0.26
4	platelets	36.10	platelets	3.64×10^−122^	mean arterial pressure	0.21
5	respiration (PaO2)	33.64	respiration (PaO2)	1.36×10^−113^	creatinine	0.20
6	respiration (FiO2)	27.17	respiration (FiO2)	1.66×10^−89^	respiration (FiO2)	0.16
7	urine output 24 hours	21.34	urine output 24 hours	2.55×10^−78^	urine output 24 hours	0.16
8	bilirubin	12.25	hypertension (HTN)	1.08×10^−30^	platelets	0.11
9	hypertension (HTN)	12.10	bilirubin	1.06×10^−29^	age	0.11
10	sex	10.10	sex	1.91×10^−22^	respiration (PaO2)	0.10
11	microorganism	8.57	microorganism	1.56×10^−16^	CAD	0.07
12	pulmonary embolism (PE)	7.83	pulmonary embolism (PE)	3.74×10^−14^	CKD with dialysis	0.07
13	initial procalcitonin (PCT)	7.29	anatomical site of infection	1.90×10^−12^	anatomical site of infection	0.06
14	anatomical site of infection	7.28	initial procalcitonin (PCT)	1.97×10^−12^	hypertension (HTN)	0.04
15	CKD with dialysis	6.09	CKD with dialysis	2.13×10^−09^	atrial fibrillation	0.04
16	diabetes mellitus (DM)	5.75	diabetes mellitus (DM)	1.36×10^−08^	CHF	0.04
17	COPD	3.66	COPD	2.67×10^−04^	cancer	0.04
18	mechanical vent	2.67	mechanical vent	7.74×10^−03^	bilirubin	0.03
19	atrial fibrillation	1.82	atrial fibrillation	6.85×10^−02^	sex	0.03
20	CKD without dialysis	1.61	CKD without dialysis	1.08×10^−01^	COPD	0.03
21	creatinine	1.38	creatinine	1.68×10^−01^	extent of infection	0.02
22	CAD	1.29	CAD	1.98×10^−01^	CKD without dialysis	0.01
23	CHF	0.47	CHF	6.38×10^−01^	pulmonary embolism (PE)	0.01
24	extent of infection	0.47	extent of infection	6.41×10^−01^	diabetes mellitus (DM)	0.01
25	cancer	0.38	cancer	7.06×10^−01^	microorganism	0.00

abs(PCC): absolute value of Pearson correlation coefficient [40]. abs(*t*-test): absolute value of Student’s *t*-test [41]. *p*-value: probability value of Student’s *t*-test. We computed each test between the target feature (vasopressors) and each feature, and then ranked the outcomes

Random Forests identified creatinine, Glasgow coma scale, mean arterial pressure, and initial procalcitonin as the most important features to identify septic shock (Table 6), that resulted in top positions also in the traditional univariate biostatistics rankings (Table 7). The Student’s *t*-tests and *p*-values identified age as the top most important feature, that instead obtained the 10^*th*^ position for the Pearson correlation coefficient (Table 7) and the 14^*th*^ position for the Random Forests ranking (Table 6).

Overall, with the significant exception of age, the Random Forests ranking and the traditional univariate biostatistics rankings showed similar positions for the features importance, confirming also the importance of the Glasgow come scale value and the blood creatinine levels to recognize patients having septic shock.

#### SOFA score feature ranking

We reported the feature ranking for SOFA score obtained by the two feature selections performed through Random Forests (Methods) in Table 8, and the feature rankings obtained through traditional biostatistics coefficients (Pearson correlation coefficient, Student’s *t*-test, *p*-values) in Table 9.

**Table 8:** SOFA score feature ranking – Random Forests

position	MDI	MDA	feature
1	9.44×10^+02^	4.35	Glasgow coma scale
2	6.88×10^+02^	2.68	creatinine
3	3.83×10^+02^	1.01	platelets
4	2.29×10^+02^	8.19×10^−01^	respiration (FiO2)
5	1.82×10^+02^	3.32×10^−01^	mean arterial pressure
6	1.59×10^+02^	6.63×10^−01^	mechanical vent
7	1.78×10^+02^	2.78×10^−01^	bilirubin
8	1.39×10^+02^	4.58×10^−01^	urine output 24 hours
9	1.73×10^+02^	1.55×10^−01^	initial procalcitonin (PCT)
10	1.14×10^+02^	1.91×10^−01^	respiration (PaO2)
11	8.34×10^+01^	5.76×10^−02^	age
12	9.38×10^+01^	3.30×10^−02^	anatomical site of infection
13	5.42×10^+01^	3.37×10^−02^	microorganism
14	1.60×10^+01^	2.26×10^−02^	chronic kidney disease (CKD) without dialysis
15	1.50×10^+01^	1.23×10^−02^	sex
16	1.44×10^+01^	9.98×10^−03^	hypertension (HTN)
17	1.26×10^+01^	9.76×10^−03^	diabetes mellitus (DM)
18	1.15×10^+01^	8.04×10^−03^	cancer
19	1.15×10^+01^	4.98×10^−03^	chronic obstructive pulmonary disease (COPD)
20	1.20×10^+01^	2.46×10^−03^	coronary artery disease (CAD)
21	8.44	4.04×10^−03^	extent of infection
22	4.45	9.73×10^−03^	chronic kidney disease (CKD) with dialysis
23	8.18	−9.64×10^−04^	congestive heart failure (CHF)
24	1.26	−1.46×10^−03^	pulmonary embolism (PE)
25	6.22	−5.16×10^−03^	atrial fibrillation

Feature ranking results obtained through random forest

**Table 9:** SOFA score features ranking – biostatistics analysis

position	abs(t) rank	abs(t)	*p*-value rank	*p*-value	abs(PCC) rank	abs(PCC)
1	age	81.88	age	1.35×10^−259^	Glasgow coma scale	0.69
2	mean arterial pressure	81.26	mean arterial pressure	7.53×10^−248^	mechanical vent	0.43
3	platelets	35.57	Glasgow coma scale	2.01×10^−155^	creatinine	0.41
4	Glasgow coma scale	34.78	platelets	1.86×10^−120^	respiration (FiO2)	0.37
5	respiration (PaO2)	32.38	respiration (PaO2)	1.63×10^−109^	urine output 24 hours	0.37
6	respiration (FiO2)	25.01	respiration (FiO2)	1.06×10^−81^	initial procalcitonin (PCT)	0.36
7	urine output 24 hours	21.21	bilirubin	7.87×10^−71^	bilirubin	0.31
8	bilirubin	21.19	urine output 24 hours	3.16×10^−66^	platelets	0.27
9	hypertension (HTN)	19.69	hypertension (HTN)	7.01×10^−60^	mean arterial pressure	0.25
10	sex	19.34	sex	1.97×10^−58^	age	0.14
11	diabetes mellitus (DM)	18.55	diabetes mellitus (DM)	4.70×10^−55^	sex	0.12
12	COPD	18.18	COPD	1.90×10^−53^	anatomical site of infection	0.10
13	mechanical vent	18.02	mechanical vent	1.04×10^−52^	CKD with dialysis	0.10
14	CAD	17.79	CAD	9.87×10^−52^	hypertension (HTN)	0.09
15	extent of infection	17.67	CHF	3.48×10^−51^	CAD	0.08
16	CHF	17.67	extent of infection	3.53×10^−51^	diabetes mellitus (DM)	0.05
17	cancer	17.66	cancer	4.01×10^−51^	CKD without dialysis	0.04
18	CKD without dialysis	17.38	CKD without dialysis	6.48×10^−50^	respiration (PaO2)	0.03
19	atrial fibrillation	17.36	atrial fibrillation	8.55×10^−50^	COPD	0.03
20	CKD with dialysis	16.92	CKD with dialysis	8.00×10^−48^	extent of infection	0.02
21	pulmonary embolism (PE)	16.78	pulmonary embolism (PE)	3.12×10^−47^	cancer	0.01
22	creatinine	14.79	creatinine	4.08×10^−42^	pulmonary embolism (PE)	0.01
23	microorganism	14.29	microorganism	3.56×10^−38^	microorganism	0.01
24	anatomical site of infection	9.65	anatomical site of infection	1.01×10^−20^	CHF	0.00
25	initial procalcitonin (PCT)	5.30	initial procalcitonin (PCT)	2.03×10^−07^	atrial fibrillation	0.00

PCC: Pearson correlation coefficient [40]. *t*-test: absolute value of Student’s *t*-test [41]. *p*-value: probability value of Student’s *t*-test. We computed each test between the target feature (SOFA score) and each feature, and then ranked the outcomes

Random Forests selected Glasgow come scale, creatinine, and platelets as most important feetures for SOFA score (Table 8). While all the biostatistics rates recognized Glasgow coma scale and platelets were recognized as relevant features too (Table 9), the Student’s *t*-test and the *p*-values ranked creatinine as 22 ^*nd*^ most important feature.

Similar to septic shock, the biostatistics techniques ranked age as a top feature, while Random Forests put it in the 11^*th*^ position of its ranking. All the other features obtained similar rank positions in all the rankings.

#### Survival feature ranking

We reported the feature ranking for survival obtained by the two feature selections performed through Random Forests (Methods) in Table 10, and the feature rankings obtained through traditional biostatistics coefficients (Pearson correlation coefficient, Student’s *t*-test, *p*-values) in Table 11.

**Table 10:** Survival feature ranking – Random Forests

position	MDI	MDA	feature
1	3.50	3.13×10^−03^	platelets
2	2.87	1.97×10^−03^	creatinine***
3	2.42	1.76×10^−03^	respiration (PaO2)***
4	2.74	9.33×10^−04^	age
5	2.74	4.53×10^−04^	initial procalcitonin (PCT)
6	1.44	1.82×10^−03^	Glasgow coma scale
7	1.99	3.79×10^−04^	respiration (FiO2)
8	7.58×10^−01^	1.62×10^−03^	mechanical vent
9	4.51×10^−01^	5.32×10^−06^	hypertension (HTN)
10	3.90×10^−01^	3.55×10^−04^	chronic obstructive pulmonary disease (COPD)
11	3.64×10^−01^	4.45×10^−04^	coronary artery disease (CAD)
12	2.39	−5.85×10^−04^	bilirubin
13	3.43×10^−01^	8.71×10^−05^	diabetes mellitus (DM)
14	4.63×10^−01^	−3.37×10^−05^	cancer
15	3.39×10^−01^	2.78×10^−04^	congestive heart failure (CHF)
16	2.16	−5.21×10^−05^	mean arterial pressure
17	2.29	−2.14×10^−03^	anatomical site of infection
18	4.93×10^−01^	−2.46×10^−04^	sex
19	3.41×10^−01^	1.75×10^−04^	urine output 24 hours
20	1.01	−1.09×10^−03^	microorganism
21	9.88×10^−02^	−5.87×10^−05^	chronic kidney disease (CKD) with dialysis
22	7.42×10^−02^	−8.02×10^−06^	pulmonary embolism (PE)
23	2.88×10^−01^	−2.28×10^−04^	extent of infection
24	3.01×10^−01^	−2.85×10^−04^	chronic kidney disease (CKD) without dialysis***
25	2.32×10^−01^	−1.09×10^−04^	atrial fibrillation

Feature ranking results obtained through Random Forests. ***: feature in a ranking position that clearly differs from its ranking positions in all the biostatistics analysis feature rankings (Table 11)

**Table 11:** Survival features ranking – biostatistics analysis

position	abs(t) rank	abs(t)	*p*-value rank	*p*-value	abs(PCC) rank	abs(PCC)
1	age	91.25	age	1.17×10^−252^	Glasgow coma scale	0.18
2	mean arterial pressure	87.57	mean arterial pressure	2.51×10^−246^	mechanical vent	0.17
3	Glasgow coma scale	79.70	Glasgow coma scale	4.76×10^−236^	bilirubin	0.17
4	pulmonary embolism (PE)	58.78	CKD with dialysis	1.24×10^−225^	platelets	0.16
5	CKD with dialysis	52.39	pulmonary embolism (PE)	4.52×10^−217^	pulmonary embolism (PE)	0.14
6	atrial fibrillation	39.53	atrial fibrillation	3.38×10^−183^	age	0.13
7	CKD without dialysis	38.98	CKD without dialysis	3.88×10^−180^	urine output 24 hours	0.13
8	platelets	36.25	cancer	1.88×10^−152^	sex	0.10
9	cancer	34.26	CHF	3.23×10^−151^	anatomical site of infection	0.09
10	CHF	34.05	extent of infection	4.91×10^−149^	CKD without dialysis	0.08
11	respiration (PaO2)	33.98	CAD	1.06×10^−140^	respiration (FiO2)	0.08
12	extent of infection	33.71	mechanical vent	2.00×10^−124^	cancer	0.07
13	CAD	32.31	platelets	1.13×10^−122^	initial procalcitonin (PCT)	0.07
14	mechanical vent	29.62	respiration (PaO2)	8.05×10^−115^	respiration (PaO2)	0.06
15	COPD	27.85	COPD	6.59×10^−114^	CHF	0.05
16	respiration (FiO2)	27.76	diabetes mellitus (DM)	7.16×10^−95^	hypertension (HTN)	0.05
17	microorganism	24.91	respiration (FiO2)	8.85×10^−92^	creatinine	0.05
18	diabetes mellitus (DM)	24.58	microorganism	7.79×10^−86^	mean arterial pressure	0.03
19	sex	19.34	sex	5.93×10^−66^	COPD	0.03
20	hypertension (HTN)	17.50	hypertension (HTN)	2.18×10^−56^	microorganism	0.03
21	anatomical site of infection	14.06	anatomical site of infection	2.44×10^−36^	atrial fibrillation	0.01
22	creatinine	10.55	creatinine	5.13×10^−23^	extent of infection	0.01
23	urine output 24 hours	9.04	urine output 24 hours	1.65×10^−18^	CAD	0.01
24	initial procalcitonin (PCT)	7.86	initial procalcitonin (PCT)	4.37×10^−14^	diabetes mellitus (DM)	0.01
25	bilirubin	0.02	bilirubin	9.83×10^−01^	CKD with dialysis	0.00

PCC: Pearson correlation coefficient [40]. *t*-test: absolute value of Student’s *t*-test [41]. *p*-value: probability value of Student’s *t*-test. We computed each test between the target feature (survival) and each feature, and then ranked the outcomes

The feature ranking results obtained for the survival target generated more divergence between Random Forests and traditional biostatistics methods, among all three target feature rankings.

Random Forests identified platelets as the most important feature (Table 10), which resulted on a top position also in the Pearson correlation coefficient ranking, but not in the ranking of the Student’s *t*-test and the ranking of the *p*-values (Table 11). Random Forests then selected creatinine, and respiration (PaO2) as most relevant features for survival, but these three features were ranked in low positions by the traditional biostatitics techniques (Table 11).

Another difference regarded chronic kidney disease (CKD) without dialysis. While the Student’s *t*-test, *p*-values, and PCC ranked this feature in mid-high positions (7^*th*^, 7^*th*^, and 11^*th*^ position, respectively) (Table 11), Random Forests considered CKD without dialysis as the penultimate less important feature (Table 10).

All the ranking methods, in this case, ranked age as a top feature.

## Discussion

Our results showed that machine learning can be employed efficiently to predict septic shock, SOFA score, and survival of patients diagnosed with sepsis, from their electronic health records data. In particular, Random Forests resulted in being the top method in correctly classifying septic shock patients, even if no method achieved good prediction performance in correctly identifying patients without septic shock (“Septic shock prediction” section) The Deep Learning model outperformed the other classifier in the SOFA score regression (“SOFA score prediction” section). Regarding the survival prediction, the Multi-Layer Perceptron Neural Network achieved the top prediction score among all the classifiers (“Survival prediction” section).

This difference in the top performing methods might be due to the different kinds and different ratios of the dataset targets (negatively imbalance for the septic shock, regression for SOFA score, and positively imbalanced for survival, “Dataset” section), and the different data processing made by each algorithm.

Regarding feature ranking, Random Forests feature selection identified several unexpected symptoms and clinical components as relevant for septic shock, SOFA score, and survival.

For septic shock, Random Forests selected creatinine as a top feature, differently from the traditional univariate biostatistics approaches (“Septic shock feature ranking” section). Recent scientific discoveries confirm this trend: the level of creatinine in the blood is often used as a biomarker for sepsis [42], especially in presence of a serious kidney injury [43].

Random Forests also ranked initial procalcitonin (PCT) as a top feature, confirming the relationship between this protein and septic shock found by Yunus and colleagues [29].

About the SOFA score prediction, the ranking positions of the Random Forests feature selection resulted in being consistent with the ranking positions of the traditional univariate biostatistics analysis. Also in this case, Random Forests also ranked initial procalcitonin (PCT) as a mid-top feature, confirming the weak positive relationship between this protein and the SOFA score found by Yunus and colleagues [29].

On the contrary, Random Forests labeled as important several features that were not ranked in top positions by the Student’s *t*-test, *p*-values, and Pearson correlation coefficient rankings. Different from the univariate biostatistics analysis, Random Forests, in fact, identified creatinine, respiration (PaO2) as top components in the classification of survived sepsis patients versus deceased sepsis patients. Kang et al. [44] recently confirmed the strong association between serum creatinine level and mortality. Regarding respiration (PaO2), Santana and colleagues [45] recently showed how the SaO2/FiO2 ratio (a rate strongly correlated to the PaO2/FiO2 ratio) is associated with mortality from sepsis. This aspect suggests the need of additional studies and analyses in this direction.

Additionally, Random Forests feature ranking showed difference with the biostatistics rankings in the last ranking positions. Random Forests, in fact, considered having chronic kidney disease (CKD) without dialysis as a scarcely important component for survival, while the traditional biostatistics rates ranked that element in top positions. Maizel and colleagues [46] confirmed our finding in 2013 by stating: “Non-dialysis CKD appears to be an independent risk factor for death after septic shock in ICU patients” [46].

## Conclusions

Sepsis is still a widespread lethal condition nowadays, and the identification of its severity can require a lot of effort. In this context, machine learning can provide effective tools to speed up the prediction of an upcoming septic shock, the prediction of the sequential organ failure, and the prediction of survival or mortality of the patient by processing large datasets in a few minutes.

In this manuscript, we presented a computational system for the prediction of these three aspects, the feature ranking of their clinical features, and the interpretation of the results we obtained. Our system consists of classifiers able to read the electronic health records of the patients diagnosed with sepsis, and to computationally predict the three targets for each of them (septic shock, SOFA score, survival) in a few minutes. Additionally, our computational intelligence system can predict the most important input features of the electronic health records of each of the three targets, again in a few minutes. We then compared the feature ranking results obtained through machine learning with the feature rankings obtained with traditional univariate biostatistics coefficients. The machine learning feature rankings highlighted the importance of some features that traditional biostatistics failed to underline. We found confirmation of the importance of these factors in the biomedical literature, which suggests the need of additional investigation on these aspects for the future.

Our discoveries can have strong implications on biomedical research and clinical practice.

First, medical doctors and clinicians can take advantage of our methods to predict survival, septic shock, and SOFA scores from any available electronic health record having the same variables of the datasets used in this study. This prediction can help doctors understand the risk of survival and septic shock for each patient, and how many organs risk to fail because of the septic episode. Doctors could use this information to decide the following steps of the therapy.

Additionally, the results of the machine learning feature ranking suggest additional, more thorough investigations on some factors of the electronic health records that would have been unnoticed otherwise: creatinine for septic shock, procalcitonin for SOFA score, and respiration (PaO2) for survival. We believe these discoveries could orientate the scientific debate regarding sepsis, and suggest to medical doctors to pay more attention to these three variables in the clinical records.

Regarding limitations, we have to report that our machine learning classifiers were unable to efficiently predict patients without septic shock among the dataset, and therefore obtained low true negative rates. We believe this drawback is due to the imbalance of the dataset, that contains 81.59% positive data instances (patients with septic shock), and only 18.41% negative data instances (patients without septic shock). In the future, we aim at exploring several over-sampling techniques to deal with this data imbalance problem [47].

Another limitation of our study was the employment of a single dataset: having an alternative dataset where to confirm our findings would make our results more robust. We looked for alternative datasets with the same clinical features to use as validation cohorts, but unfortunately could not find them. Because of this issue and of the small size of our dataset (364 patients), we cannot confirm that our approach is generalizable to other cohorts.

In the future, we plan to employ alternative methods for feature ranking, to compare their results with the results we obtained through Random Forests. We also plan to employ similarity measures to analyze the semantic similarity between patients [48].

## Supplementary Information

**Additional file 1** Supplementary information containing details regarding data engineering, algorithms, and metrics employed in the analysis.

## Abbreviations

AUC: Area under the curve
BIDMC: Beth Israel deaconess medical center
CAD: Coronary artery disease
CHF: Congestive heart failure
CKD: Chronic kidney disease
COPD: Chronic obstructive pulmonary disease
DM: Diabetes mellitus
DT: Decision tree
EE: Error estimation
EHRs: Electronic health records
FiO2: Respiration
FR: Feature ranking
HTN: Hypertension
Inc.: Incorporation
MAP: Mean arterial pressure
MCC: Matthews correlation coefficient
MDA: Mean decrease in accuracy
MDI: Mean decrease in impurity
MIMIC-III: Multiparameter intelligent monitoring in intensive care III
MLP: Multilayer perceptron
MS: Model selection
NPV: Negative predictive value
NICU: Neonatal intensive care unit
NN: Neural network
PaO2: Respiration
PCC: Pearson correlation coefficient
PCT: Procalcitonin
PE: Pulmonary embolism
PPV: Positive predictive value
R^2^: Coefficient of determination
SOFA: Sequential organ failure assessment
SVM: Support vector machine
