# Supplementary material for: Data analytics and clinical feature ranking of medical records of patients with sepsis
Source: BioData Min. 2021 Feb 3;14:12. doi: 10.1186/s13040-021-00235-0 (PMC7860202; doi:10.1186/s13040-021-00235-0)
Supplement: Supplementary file 1 — Additional file 1 Supplementary information containing details regarding data engineering, algorithms, and metrics employed in the analysis. [file 13040_2021_235_MOESM1_ESM.pdf]

S7 Supplementary information

S7.1 Data engineering

By mapping the values of the features to numbers, we added some values to the original dataset to represent data instances of multiple cases.

For anatomical site of infection, the original dataset [29] had 7 possible values: 1 = pneumonia; 2 = urinary tract infection; 3 = musculoskeletal infection; 4 = brain; 5 = heart; 6 = gastrointestinal infection; 7 = unspecified. To these original values, we added elements representing infections on multiple sites: 9 = (1 and 6); 10 = (1, 2 and 3); 11 = (1 and 3); 12 = (1 and 4); 13 = (2 and 3); 14 = (3 and 6); 15 = (6 and 1); 16 = (7 and 2).

For extent of infection, the original dataset [29] had 2 possible values: 1 = bacteremia, and 2 = focal infection. To these original values, we added an element representing both extents of infection: 3 = (1 and 2).

For microorganism, the original dataset [29] had 4 possible values: 1 = gram positive; 2 = gram negative; 3 = unspecified; 4 = viral. To these original values, we added an element representing multiple microorganism conditions: 5 = (1 and 2); 6 = (2 and 3).

As already explained in the Dataset section, we derived the values of the survival feature from the original outcome feature [29]. To each patient having original outcome = 1 (the patient improves and is discharged) or outcome = 4 (the patient is admitted to or transferred to the intensive care unit, improves and is subsequently discharged), we assigned the value survival = 1, meaning survived To each patient having original outcome = 2 (the patient dies in the ward floor) or outcome = 3 (the patient is admitted to or transferred to the intensive care unit and dies), instead we assigned the value survival = 0, meaning deceased.

We changed the values of the original Vasopressors feature, that indicates the septic shock, this way: for no septic shock, we replaced 2 with 0, and for septic shock we kept 1.

In the original dataset, the sepsis outcome has four possible values [29]:

1 = The patient improved and was discharged;

2 = The patient deceased on the ward floor;

3 = The patient was transferred to the intensive care unit (ICU), but then deceased;

4 = The patient was transferred to the ICU, improved, and was subsequently discharged.

We set our newly introduced survival feature equal to 1 (survival = true) for patients previously listed with sepsis outcome 1 or 4, and we set it equal to 0 (survival = false) for patients previously listed with sepsis outcome 2 or 3. We then removed the original feature outcome.

We also removed the “length of stay (LOS)” feature, because it can be an indicator of sepsic severity itself.

S7.2 Methods

Let us consider the now-classical supervised learning framework [48]. Let $\mathcal{X} = \mathcal{X}_1 \times \cdots \times \mathcal{X}_f$ be the input space, consisting of n_f features, and let $\mathcal{Y}$ be the output space.

$\mathcal{X}_i$, with $i \in \{1, \cdots, n_f\}$, can be a categorical feature space (the values of the features belong to a finite unsorted set) or a numerical-valued feature space (the values of the features belong to a possibly infinite sorted set). In the case of categorical feature space with more than two categories, we opt for the one hot encoding and we map it in a numerical feature space [49].

If $\mathcal{Y} \subseteq \mathbb{R}$ the problem under exam is a regression task while if $\mathcal{Y} \subseteq \{0, 1\}$ the problem under exam is a binary classification problem, conventionally we will indicate with 1 a positive outcome and with 0 a negative outcome. Let $\mathcal{D}_n = \{(X_1, Y_1), \dots, (X_n, Y_n)\}$, where $X_i \in \mathcal{X}$ and $Y_i \in \mathcal{Y}$ $\forall i \in \{1, \cdots, n\}$, be a sequence of $n \in \mathbb{N}^*$ samples drawn independently from an unknown probability distribution μ over $\mathcal{X} \times \mathcal{Y}$.

Note that some values of X may be missing [50]. In this case, if the missing value is in a categorical feature, an additional category for missing values is introduced for that feature. If, instead, the missing value is associated with a numerical feature the missing value is replaced with the mean value of that feature and an additional logical feature is introduced to indicate whether the value of that feature is missing or not for a particular sample. Let us consider a model (function) $f : \mathcal{X} \to \mathcal{Y}$ chosen from set $\mathcal{F}$ of possible hypotheses.

An algorithm $\mathscr{A}_{\mathcal{H}} : \mathcal{D}_n \times \mathcal{F} \to f$ characterized by its hyper-parameters $\mathcal{H}$ selects a model inside a set of possible ones based on the available dataset (subsection S7.3). The error of f in approximating the performance $\mathbb{P}\{Y \mid X\}$ is measured by a prescribed metric $M : \mathcal{F} \to \mathbb{R}$. Note that there are many different metrics available in literature for both classification and regression [49] (Supplementary information).

Note also that, for binary classification problems, $\mathcal{D}_n$ may be imbalanced (namely the $|\{(X, Y) \in \mathcal{D}_n : Y = 0\}|$ may be $\gg$ or $\ll$ than the $|\{(X, Y) \in \mathcal{D}_n : Y = 1\}|$) and this may result in classifiers which produce unsatisfactory results on one of the two classes resulting in unsatisfactory metrics performance [51]; for this reason we discuss the problem and show how we tackle it in this work (subsection S7.4).

To tune the performance of the $\mathscr{A}_{\mathcal{H}}$, namely to select the best set of hyper-parameters, and to estimate the performance of the final model according to the desired metrics, Model Selection (MS) and Error Estimation (EE) phases [52] need to be performed (subsection S7.5).

Finally, we will also check for possible spurious correlations in the data by performing the Feature Ranking phase [53]. In fact, once the model is built based on the different learning algorithms and has been confirmed to be a sufficiently accurate representation of the $\mathbb{P}\{Y \mid X\}$ during the EE phase, one has to investigate how and how much the model is affected by the different features that have been exploited to build the model itself during the feature ranking procedure (subsection S7.6).

We provide more details about the implementation of our methods in the Supplementary Information.

S7.3 Algorithm details

In this section we briefly recall the four algorithms what we have exploited in this study by pointing out the idea behind them, how to use them, and their hyper-parameters.

The setting of the application of all these algorithms is the supervised machine learning framework: supervised binary classification for the prediction of survival and septic shock, and supervised regression for the prediction of SOFA score.

The selected algorithms represent the most effective algorithms in four families of methods [54]: rule based methods, ensemble methods, kernel methods, and neural networks.

S7.3.1 Decision Tree

A binary Decision Tree (DT) [55] belongs to the family of the rule based methods. The DT is a flowchart-like structure in which each internal node represents a test of a feature, each branch represents the outcome of the test, and each leaf node represents an output of the tree. A path from the root to a leaf represents a model rule. A DT is built with a recursive schema until it reaches its desired depth d, which is the DT hyper-parameter that needs to be tuned during the MS phase. Each node of the DT, starting from the root node, is built by choosing the attribute and the cut that most effectively split the set of samples into two subsets based on the information gain. The decision trees can be exploited both for classification and regression; they handle categorical features, numerical features, and missing values well, and they do not suffer from numerical issues (no normalization of the data is needed).

S7.3.2 Random Forests

The Random Forests (RF) [56] belong to the family of the ensemble methods. RF combine bagging to random subset feature selection. In bagging, each tree is independently constructed using a bootstrap sample of the dataset. RF add an additional layer of randomness to bagging. In addition to constructing each tree using a different bootstrap sample of the data, RF change how the classification trees are constructed. In standard trees, each node is split using the best division among all variables. In a RF, each node is split using the best among a subset of predictors randomly chosen at that node. Eventually, a simple majority vote is taken for prediction. The accuracy of the final model depends mainly on three different factors: how many trees compose the forest, the accuracy of each tree and the correlation between them. The accuracy for RF converges to a limit as the number of trees n_t in the forest increases, while it rises as the accuracy of each tree increases and the correlation between them decreases. There are several hyper-parameters which characterise the performance of the final model: the number of trees, the number of samples to extract during the bootstrap procedure, the depth of each tree, the number of predictors exploited in each subset during the growth of each tree, and finally the weights assigned to each tree. Nevertheless, in common applications, the RF sensitivity to these factors is quite low [56]. Since RF is basically a combination of many DTs, RF can be exploited both for classification and regression. Random Forests handle categorical features, numerical features, and missing values well, and they do not suffer from numerical issues (no normalization of the data is needed).

S7.3.3 Support Vector Machines

The Support Vector Machines (SVM) [57] belong to the family of the kernel methods. Kernel methods are a family of techniques which exploits the "kernel trick" for distances in order to extend linear techniques to the solution of non-linear problems [58]. Kernel methods select the model which minimizes the trade-off between the performance, measured with a defined metric (Supplementary information), over the data and the complexity of the solution, measured with different measures of complexities [57, 54]. Support Vector Machines (SVM), linear SVM (linear) and non-linear SVM (kernel), represent the most known and effective Kernel methods techniques. The hyper-parameters of the SVM are: the kernel, which is usually fixed and is the linear one for SVM (linear) and the Gaussian one for SVM (kernel) because of the reasons described in [59], the kernel hyper-parameter γ for SVM (kernel) and the regularization hyper-parameter C. C and γ need to be tuned during the MS phase. SVM are able to handle directly both binary classification and regression. They do not handle categorical features directly (consequently the one hot [49] is needed) and they do suffer from numerical issues and consequently data must be re-scaled (in our case all the numerical features and targets have been scaled to have zero mean and variance equal to one).

S7.3.4 Multi-Layer Perceptron Neural Network with dropout

The Multilayer Perceptron Network with Dropout (MLP) [60, 31] belongs to the family of the neural networks. Neural networks are a family of techniques which combine together many simple models of a human brain neuron, called perceptrons [61], in order to build a complex network. The neurons are organized into stacked layers, connected together by weights that are learned based on the available data via backpropagation [62]. If the architecture of the neural networks consists of only one hidden layer, it is called shallow, while, if multiple layers are staked together, the architecture is defined as deep. From a functional point of view both architecture have the same representation power [63] but in practice, for some applications like natural language processing and image analysis, deep networks outperform the shallow ones [31]. In our context, instead, where the number of samples and features is limited, it is more reasonable to use a shallow network [60, 31]. In particular, in this work, we exploited a pretty well known and effective architecture, the MLP, where a single hidden layer is present, we train it with adaptive subgradient methods, and we tuned the following hyper-parameters during the MS phase [31]: the number of neurons in the hidden layer n_h, the dropout rate p_d, the percentage of data to use as batch size p_b, the learning rate r_l, the fraction of gradient to keep at each step ρ, the learning rate decay r_d, and the activation function. The MLP is able to directly handle both classification and regression. Instead, like SVM, they do not handle categorical features directly (consequently the one hot [49] is needed) and they do suffer from numerical issues and consequently the data must be re-scaled (in our case we exploited the same re-scaling method exploited for SVM).

S7.3.5 Naive Bayes

Naive Bayes (NB) method [64] is a classification algorithms based on applying the theorem of Bayes with the naive assumption of conditional independence between

every pair of features given the value of the class variable. In spite of its apparently over-simplified assumptions, NB classifiers have worked quite well in many real-world situations since they require a small amount of training data to estimate the necessary parameters. Moreover, NB learners and classifiers can be extremely fast compared to more sophisticated methods.

S7.3.6 k-nearest neighbors

The k-nearest neighbors (k-NN) algorithm [65] is a simple and easy-to-implement supervised machine learning algorithm that can be used to solve both classification and regression problems. The k-NN algorithm assumes that similar things exist in close proximity. In other words, similar things are near to each other. Then k-NN then suggests, to label a point, to search for the k closest points in the training set ad use the mode of their k labels. k is an hyperparameter that need to be tuned during the MS phase.

S7.3.7 Logistic regression

Logistic regression (LR) algorithm [66] is quite similar to the linear SVM (namely same structure and hyperperparameters), the only difference is in the scoring function which is actually converted into a probability thanks to the use of the sigmoid function. It is quite often exploited in classification tasks because of its intrinsic ability to provide both class membership and confidence.

S7.3.8 Deep learning model with weight decay

Deep Learning (DL) algorithms [67] for vectorial data, like the one we have in this paper, consists in an MLP with many layers. In this work, for completeness, we decided to insert in addition to the MLP also a 3 layered MLP with weight decay. The number of neurons in each layer, respectively l_1, l_2, and l_3 are hyperparameters to be tuned. Moreover, the amount of weight decay wd, which acts as a regulariser similar to the one of SVM and LR, need also to be tuned, together with the other regularisers during the MS phase.

S7.4 Handling unbalanced datasets in binary classification

Data available in bioinformatics for binary classification are often strongly unbalanced [68, 69, 70]. However, most learning algorithms do not work well with imbalanced datasets and tend to poorly perform on the minority class and for these reasons several techniques have been developed in order to address this issue [51].

The first step toward the solution of this problem is to avoid applying the inappropriate evaluation metrics for model generated using imbalanced data [71]. For example, overall accuracy is a very dangerous metric in this context since the more unbalanced is the dataset the more this metric tends to promote models which poorly perform on the minority class. For this reason, in this study we also included other metrics like Precision/Specificity, Recall/Sensitivity, F_1 score, MCC, and AUC which are more suited for the case of imbalanced data (Supplementary information).

The second step toward the mitigation of the effects of having an unbalanced dataset is to modify the algorithm or the data, but currently the most practical

and effective method involves the re-sampling of the data in order to synthesize a balanced dataset [51]. For this purpose we can under- or over-sample the dataset. Under-sampling balances the dataset by reducing the size of the abundant class. By keeping all samples in the rare class and randomly selecting an equal number of samples in the abundant class, a new balanced dataset can be retrieved for further modelling. Note that this method wastes a lot of information (many samples may not be used). For this reason the oversampling strategy is more often exploited. It tries to balance the dataset by increasing the size of rare samples, and makes both the positive class and the negative class have the same distribution. Rather than removing abundant samples, new rare samples are generated (for example by repetition, by bootstrapping, or by synthetic minority). The latter method is the one that we exploited in this paper.

S7.5 Model selection and error estimation

MS and EE deal with the problem of tuning and assessing the performance of a learning algorithm [52]. Resampling techniques like k-fold cross validation and non-parametric bootstrap are often used by practitioners because they work well in many situations [72]. Other alternatives exist, which represent bases in the Statistical Learning Theory and give more insight into the learning process. Examples of methods in this last category are: the seminal work of the Vapnik-Chervonenkis Dimension, its improvement with the Rademacher Complexity, the theory of compression, the Algorithmic Stability breakthrough, the PAC-Bayes theory, and more recently the Differential Privacy theory [52].

In this work we will exploit the resampling techniques which rely on a simple idea: the original dataset $\mathcal{D}_n$ is resampled once or many (n_r) times, without replacement, to build three independent datasets called learning, validation and test sets, respectively $\mathcal{L}_l^r$, $\mathcal{V}_v^r$, and $\mathcal{T}_t^r$, with $r \in \{1, \cdots, n_r\}$. Note that $\mathcal{L}_l^r \cap \mathcal{V}_v^r = \oslash$, $\mathcal{L}_l^r \cap \mathcal{T}_t^r = \oslash$, $\mathcal{V}_v^r \cap \mathcal{T}_t^r = \oslash$, and $\mathcal{L}_l^r \cup \mathcal{V}_v^r \cup \mathcal{T}_t^r = \mathcal{D}_n$ for all $r \in \{1, \cdots, n_r\}$.

Then, in order to select the best combination of the hyper-parameters $\mathcal{H}$ in a set of possible ones $\mathfrak{H} = \{\mathcal{H}_1, \mathcal{H}_2, \cdots\}$ for the algorithm $\mathscr{A}_{\mathcal{H}}$ or, in other words, to perform the MS phase, the following procedure has to be applied:

$$\mathcal{H}^* : \quad \arg\min_{\mathcal{H} \in \mathfrak{H}} \sum_{r=1}^{n_r} M(\mathscr{A}_{\mathcal{H}}(\mathcal{L}_l^r), \mathcal{V}_v^r), \tag{1}$$

where $\mathscr{A}_{\mathcal{H}}(\mathcal{L}_l^r)$ is a model built with the algorithm $\mathscr{A}$ with its set of hyper-parameters $\mathcal{H}$ and with the data $\mathcal{L}_l^r$ and where $M(f, \mathcal{V}_v^r)$ is a desired metric. Since the data in $\mathcal{L}_l^r$ are independent from the ones in $\mathcal{V}_v^r$, the idea is that $\mathcal{H}^*$ should be the set of hyper-parameters which allows to achieve a small error on a data set that is independent from the training set.

Then, in order to evaluate the performance of the optimal model which is $f_{\mathscr{A}}^* = \mathscr{A}_{\mathcal{H}^*}(\mathcal{D}_n)$ or, in other words, to perform the EE phase, the following procedure has to be applied:

$$M(f_{\mathscr{A}}^*) = \frac{1}{n_r} \sum_{r=1}^{n_r} M(\mathscr{A}_{\mathcal{H}^*}(\mathcal{L}_l^r \cup \mathcal{V}_v^r), \mathcal{T}_t^r). \tag{2}$$

Since the data in $\mathcal{L}_l^r \cup \mathcal{V}_v^r$ are independent from the ones in $\mathcal{T}_t^r$, $M(f_{\mathscr{A}}^*)$ is an unbiased estimator of the true performance, measured with the metric M, of the final model [52].

If $n_r = 1$, if l, v, and t are aprioristically set such that $n = l + v + t$, and if the resample procedure is performed without replacement, the hold out method is obtained [52]. For implementing the complete nested k-fold cross validation, instead, it is needed to set $n_r \leq \binom{n}{k}\binom{n-\frac{n}{k}}{k}$, $l = (k-2)\frac{n}{k}$, $v = \frac{n}{k}$, and $t = \frac{n}{k}$ and the resampling must be done without replacement [72]. Finally, for implementing the nested non-parametric bootstrap, $l = n$ and $\mathcal{L}_l^r$ must be sampled with replacement from $\mathcal{D}_n$, while $\mathcal{V}_v^r$ and $\mathcal{T}_t^r$ are sampled without replacement from the sample of $\mathcal{D}_n$ that have not been sampled in $\mathcal{L}_l^r$ [72]. Note that for the bootstrap procedure $n_r \leq \binom{2n-1}{n}$. In this paper the complete nested k-fold cross validation (with $k = 10$) is exploited because it represents the state-of-the-art approach [72, 52].

S7.6 Feature ranking

Once the models are built and have been confirmed to be sufficiently effective in predicting the desired quantities, we decided to investigate how these models are affected by the different features used in the model identification phase in order to understand if the models also have a foundation which relies on the underline phenomena or if the model just captures spurious correlations [53]. This procedure is called feature ranking (FR) and allows to detect if the importance of those features, that are known to be relevant from a physical perspective, are appropriately taken into account by the learned models. The failure of the computational model to properly account for the relevant features might indicate poor quality in the measurements or spurious correlations. FR therefore represents an important step of model verification, since it should generate consistent results with the available knowledge of the phenomena under exam.

In this section, we first describe the machine learning methods scope (subsubsection S7.6.1) and then the traditional univariate biostatistics techniques (subsubsection S7.6.2) we used for this task.

S7.6.1 Random Forests feature ranking

In this context, feature rankings methods based on Random Forests are aiming the most effective machine learning techniques [73, 74], particularly in the context of bioinformatics [75, 76] and health informatics [4]. Several measures are available for feature importance in Random Forests. One approach is the one based on the Gini Importance or Mean Decrease in Impurity (MDI) which calculates each feature importance as the sum over the number of splits (across all trees) that include the feature, proportionally to the number of samples it splits. Another powerful approach is the one based on the Permutation Importance or Mean Decrease in Accuracy (MDA), where the importance is assessed for each feature by removing the association between that feature and the target. This is achieved by randomly permuting [77] the values of the feature and measuring the resulting increase in error. The influence of the correlated features is also removed. In details, for every tree, two quantities are computed: the first one is the error on the out-of-bag samples as they are used during prediction, while the second one is the error on the out-of-bag samples after a random permutation of the values of a variable. These two

values are then subtracted and the average of the result over all the trees in the ensemble is the raw importance score for the variable under exam. Both MDI and MDA can be adopted since they can be easily carried out during the main prediction process inexpensively.

Despite the effectiveness of MDI and MDA, when the number of samples is small, these methods may be unstable [78, 79, 80]. For this reason, in this work, instead of running the Feature Ranking (FR) procedure just once, analogously to what we have done for MS and EE, we sub-sample $\mathcal{D}_n$ such that $\mathcal{S}_m \subset \mathcal{D}_n$ with $m = \mid \mathcal{S}_m \mid = p_{\text{FR}} n$, namely we randomly sample without replacement $100 \cdot p_{\text{FR}}\%$ of the data in $\mathcal{D}_n$, we perform the FR using $\mathcal{S}_m$ and we repeat the procedure n_{FR} times. The final rank of a feature will be the mode of its ranking position in the different repetitions of the ranking position and the MDI and MDA are the median value over the different repetitions.

We then aggregated the two rankings into a unified merged ranking by using Borda's method [81], where we summed the two position of each feature in the two rankings, and sorted the ranking accordingly.

S7.6.2 Biostatistics feature ranking

To further validate and assess the results we obtained through Random Forests, we applied traditional univariate biostatistics rates for feature ranking, though the Student's t-test, the analysis of its p-value, and the Pearson correlation coefficient.

The Student's t-test is a statistical test that verifies if the expected value of a variable Student's t-distribution relates to the expected value of another variable distribution, or not [82]. A low absolute t score means low correlation between the feature and the target, and a high absolute t score means high correlation. The p-value of the Student's t-test represents the probability that the result achieved by the test came by chance. p-values range between 0 and 1, where a low score means high significance, and a high score means insignificance.

In our biostatistics analysis, we also use the Pearson correlation coefficient (PCC) [83]. PCC is a measure of correlation between two numerical lists, its score is $+1$ for two identical lists, 0 for two lists with no correlation, and -1 for two lists with total linear opposite correlation.

We employed these univariate statistical rates to infer the relationship of each clinical feature with each target feature (septic shock, survival, SOFA score) independently from the other clinical factors.

S7.7 Metrics

For what concerns the metrics $M(f)$ exploited for evaluating the performance of a model f learned from the data based on the methods described above, we have to recall different metrics based on the different tasks under exam (binary classification or regression) [49]. In order to define them let us first consider a subset of the available data $\mathcal{T}_m \subseteq \mathcal{D}_n$ where $m = \mid \mathcal{T}_m \mid \leq n$.

S7.7.1 Metrics for binary classification

For binary classification many different metrics are available in literature. In this paper, we report the most common ones. Let us define the True Positive (TP), True

Negative (TN), False Positive (FP), and False Negative (FN), on this data as

$$\mathrm{TP}(f) = \sum_{(X,Y)\in\mathcal{T}_m:Y=1} \mathbb{1}\{f(X)=1\} \quad (3)$$
$$\mathrm{TN}(f) = \sum_{(X,Y)\in\mathcal{T}_m:Y=0} \mathbb{1}\{f(X)=0\} \quad (4)$$
$$\mathrm{FN}(f) = \sum_{(X,Y)\in\mathcal{T}_m:Y=1} \mathbb{1}\{f(X)=0\} \quad (5)$$
$$\mathrm{FP}(f) = \sum_{(X,Y)\in\mathcal{T}_m:Y=0} \mathbb{1}\{f(X)=1\} \quad (6)$$

Then we can define the five metrics exploited in this paper for binary classification models

$$\mathrm{MCC}(f) = \frac{\mathrm{TP}(f)\cdot\mathrm{TN}(f) - \mathrm{FP}(f)\cdot\mathrm{FN}(f)}{\sqrt{(\mathrm{TP}(f)+\mathrm{FP}(f))\cdot(\mathrm{TP}(f)+\mathrm{FN}(f))\cdot(\mathrm{TN}(f)+\mathrm{FP}(f))\cdot(\mathrm{TN}(f)+\mathrm{FN}(f))}} \quad (7)$$
$$\mathrm{F}_1\ \mathrm{score}(f) = \frac{2\cdot\mathrm{TP}(f)}{2\cdot\mathrm{TP}(f)+\mathrm{FP}(f)+\mathrm{FN}(f)}, \quad (8)$$
$$\mathrm{accuracy}(f) = \frac{\mathrm{TP}(f)+\mathrm{TN}(f)}{\mathrm{TP}(f)+\mathrm{FN}(f)+\mathrm{TN}(f)+\mathrm{FP}(f)} \quad (9)$$
$$\mathrm{TP\ rate}(f) = \frac{\mathrm{TP}(f)}{\mathrm{TP}(f)+\mathrm{FN}(f)} \quad (10)$$
$$\mathrm{TN\ rate}(f) = \frac{\mathrm{TN}(f)}{\mathrm{TN}(f)+\mathrm{FP}(f)}, \quad (11)$$
$$\mathrm{PPV}\ (f) = \frac{\mathrm{TP}(f)}{\mathrm{TP}(f)+\mathrm{FP}(f)} \quad (12)$$
$$\mathrm{NPV}\ (f) = \frac{\mathrm{TN}(f)}{\mathrm{TN}(f)+\mathrm{FN}(f)} \quad (13)$$
$$\mathrm{PR\ AUC}(f) = \text{Area Under the Curve of the Precision and Recall curve} \quad (14)$$
$$\mathrm{ROC\ AUC}(f) = \text{Area Under the Receiver Operating Characteristics Curve} \quad (15)$$

where the Matthews Correlation Coefficient is indicated with MCC. In our results, we give more importance to the scores of MCC because it is the only binary evaluation rate that takes into account the ratio of the negative set and positive set [35, 34].

S7.7.2 Metrics for regression

Also for regression many different metrics are available in literature and we report the most common ones exploited for regression models: the Root Mean Square Error (RMSE), the Mean Absolute Error (MAE), the Mean Square Error (MSE), the Symmetric Mean Absolute Percentage Error (SMAPE), and the Coefficient of

Determination (R^2). They are defined as follows

$$\mathrm{RMSE}(f) = \sqrt{\frac{1}{m}\sum_{(X,Y)\in\mathcal{T}_m}(f(X)-Y)^2}, \tag{16}$$

$$\mathrm{MAE}(f) = \frac{1}{m}\sum_{(X,Y)\in\mathcal{T}_m} \mid f(X)-Y \mid, \tag{17}$$

$$\mathrm{MSE}(f) = \frac{1}{m}\sum_{(X,Y)\in\mathcal{T}_m}(f(X)-Y)^2, \tag{18}$$

$$\mathrm{SMAPE}(f) = \frac{100\%}{m}\sum_{(X,Y)\in\mathcal{T}_m}\frac{|f(X)-Y|}{\frac{|f(X)|+|Y|}{2}}, \tag{19}$$

$$\mathrm{R}^2(f) = \frac{\sum_{(X,Y)\in\mathcal{T}_m} f(X)-\bar{Y}}{\sum_{(X,Y)\in\mathcal{T}_m} f(X)-Y}, \quad \bar{Y} = \frac{1}{m}\sum_{(X,Y)\in\mathcal{T}_m} Y. \tag{20}$$

S7.8 Barcharts

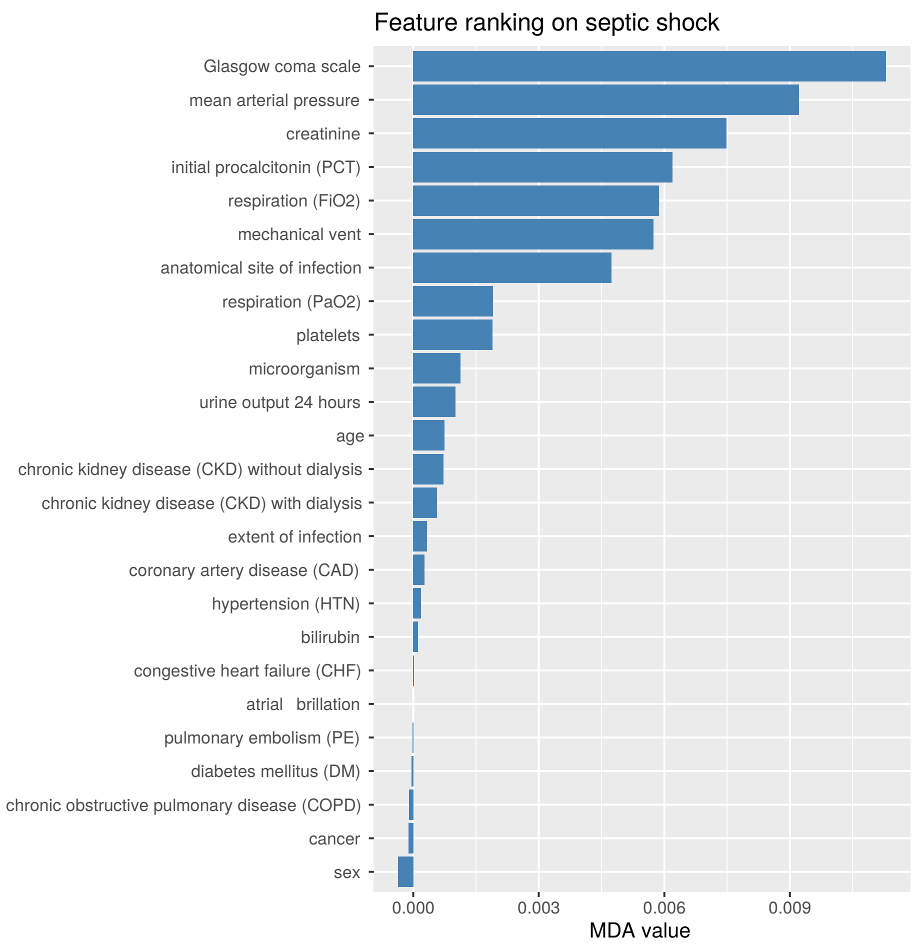

Figure S2: **Random Forests MDA Feature Ranking for septic shock.**

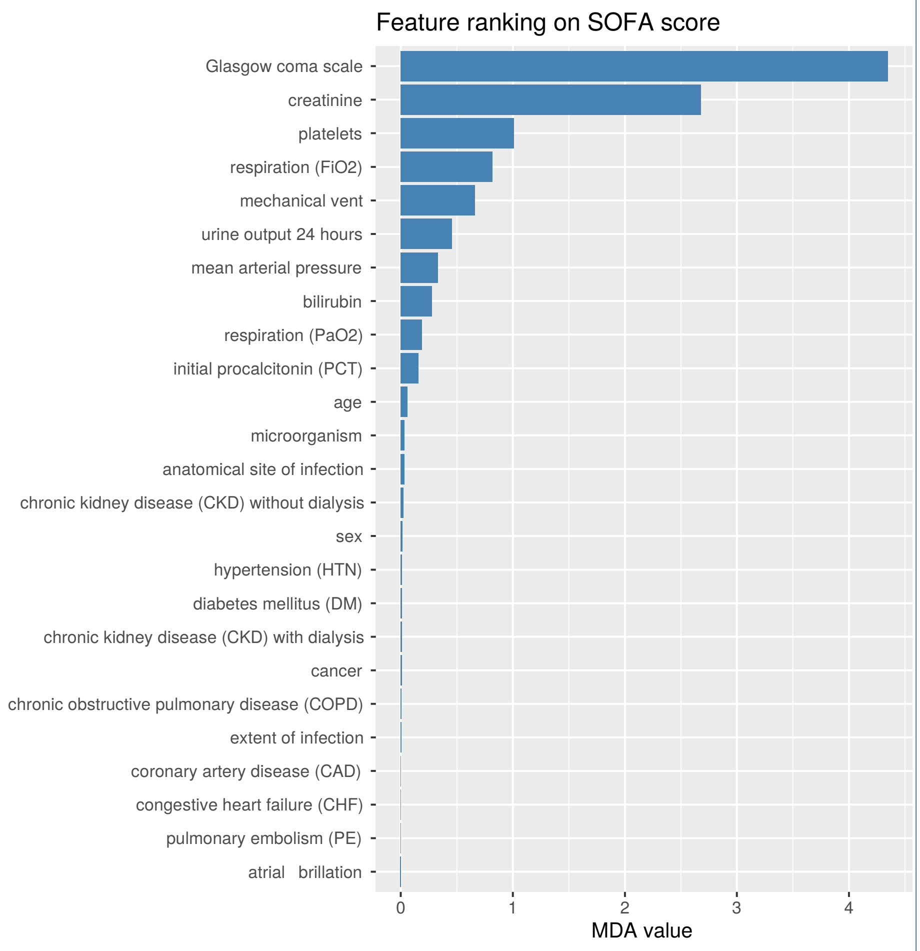

Figure S3: **Random Forests MDA Feature Ranking for SOFA score.**

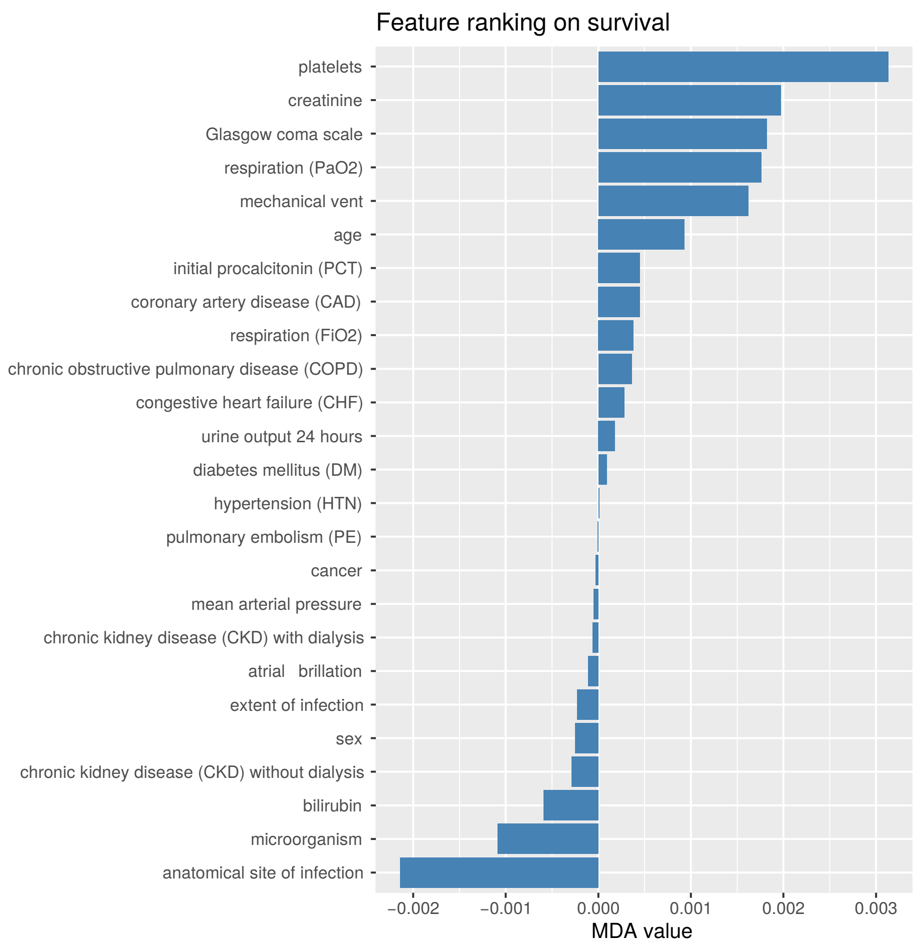

Figure S4: **Random Forests MDA Feature Ranking for survival**.
